# Downregulation of growth plate genes involved with the onset of femoral head separation in young broilers

**DOI:** 10.3389/fphys.2022.941134

**Published:** 2022-08-08

**Authors:** Adriana Mércia Guaratini Ibelli, Jane de Oliveira Peixoto, Ricardo Zanella, João José de Simoni Gouveia, Maurício Egídio Cantão, Luiz Lehmann Coutinho, Jorge Augusto Petroli Marchesi, Mariane Spudeit dal Pizzol, Débora Ester Petry Marcelino, Mônica Corrêa Ledur

**Affiliations:** ^1^ Embrapa Suínos e Aves, Concórdia, Brazil; ^2^ Programa de Pós-Graduação em Ciências Veterinárias, Universidade Estadual do Centro-Oeste, Guarapuava, Brazil; ^3^ Universidade de Passo Fundo, Passo Fundo, Brazil; ^4^ Universidade Federal do Vale do São Francisco, UNIVASF, Petrolina, Brazil; ^5^ Laboratório de Biotecnologia Animal, Escola Superior de Agricultura “Luiz de Queiroz”, Universidade de SP, Piracicaba, Brazil; ^6^ Departamento de Genética, Faculdade de Medicina de Ribeirão Preto, Universidade de SP, Ribeirão Preto, Brazil; ^7^ Programa de Pós-Graduação Em Zootecnia, Universidade do Estado de SC, UDESC-Oeste, Chapecó, Brazil; ^8^ Faculdade de Concórdia FACC, Concórdia, Brazil

**Keywords:** chickens, gene expression, FBN2, FHS, femoral head necrosis, RNA-seq

## Abstract

Femoral head separation (FHS) is characterized by the detachment of growth plate (GP) and articular cartilage, occurring in tibia and femur. However, the molecular mechanisms involved with this condition are not completely understood. Therefore, genes and biological processes (BP) involved with FHS were identified in 21-day-old broilers through RNA sequencing of the femoral GP. 13,487 genes were expressed in the chicken femoral head transcriptome of normal and FHS-affected broilers. From those, 34 were differentially expressed (DE; FDR ≤0.05) between groups, where all of them were downregulated in FHS-affected broilers. The main BP were enriched in receptor signaling pathways, ossification, bone mineralization and formation, skeletal morphogenesis, and vascularization. RNA-Seq datasets comparison of normal and FHS-affected broilers with 21, 35 and 42 days of age has shown three shared DE genes (*FBN2*, *C1QTNF8*, and *XYLT1*) in GP among ages. Twelve genes were exclusively DE at 21 days, where 10 have already been characterized (*SHISA3, FNDC1, ANGPTL7, LEPR, ENSGALG00000049529, OXTR, ENSGALG00000045154, COL16A1, RASD2, BOC, GDF10*, and *THSD7B*). Twelve SNPs were associated with FHS (*p* < 0.0001). Out of those, 5 were novel and 7 were existing variants located in 7 genes (*RARS, TFPI2, TTI1, MAP4K3, LINK54*, and *AREL1)*. We have shown that genes related to chondrogenesis and bone differentiation were downregulated in the GP of FHS-affected young broilers. Therefore, these findings evince that candidate genes pointed out in our study are probably related to the onset of FHS in broilers.

## Introduction

Leg problems are considered one of the main issues in the chicken production (Li et al., 2015). The occurrence of these disorders in commercial flocks was estimated up to 30% depending on age, density, within others, having a huge impact on animal health and welfare (Knowles et al., 2008; Federici et al., 2016). Femoral head necrosis (FHN) can affect up to 75 and 88% of commercial broilers with 28 and 42 days of age, respectively, being reported as one of the major bone pathologies (McNamee and Smyth, 2000; Durairaj et al., 2009; Wideman, 2016; Liu K. et al., 2021). Some authors classified FHN depending on its severity, such as femoral head separation (FHS), when the growth plate is separated from cartilage and femoral head separation with growth plate lacerations (FHSL), when there are lesions in the femoral growth plate (Li et al., 2015; Packialakshmi et al., 2015). This condition is also known as bacterial chondronecrosis with osteomyelitis (BCO) when it is associated with bacterial infection (Jiang et al., 2015; Al-rubaye et al., 2017; Ramser et al., 2021).

The FHS is a multifactorial disorder, which could be caused by traumatic or non-traumatic factors (Liu K. et al., 2021). It is characterized by a degenerative skeletal problem presenting focal cell death, fibrotic thickening, and atrophic changes in the cartilage (Packialakshmi et al., 2015). Furthermore, some authors consider FHS as a primary cartilage defect, since reduced chondrocyte density was observed in the GP of affected broilers (Wilson et al., 2019). These failures lead to cartilage degeneration and necrosis, which predispose animals to develop FHN (Packialakshmi et al., 2015; Wilson et al., 2019). The growth plate chondrocytes are required to the proper development and growth of endochondral bones (Hallett et al., 2019). The disruption in homeostasis of bone formation and resorption through the imbalance of catabolic and anabolic factors (Wnt signaling, HIF expression, extracellular matrix production, within others) was described in broilers with 42 and 56 days of age (Yu et al., 2020). Therefore, the understanding of the GP molecular mechanisms is essential to clarify the genes responsible for triggering FHS/FHN in chickens.

Over the years, global gene expression studies have provided a better understanding of the putative acting genes in the two tissues that are the primary sites of femoral head disorders. From transcriptomes of the femur articular cartilage (AC) and growth plate (GP) of 35-day-old broilers (Peixoto et al., 2019; Hul et al., 2021; Goldoni et al., 2022), several candidate genes were prospected. Oliveira et al. (2020) also pointed out that osteochondral downregulated genes are potentially triggering BCO in tibia of 42-day-old broilers. Most of the studies with FHN/FHS in chickens are focused on understanding the ossification processes and, generally, in animals older than 35 days of age (Li et al., 2015; Paludo et al., 2017; Peixoto et al., 2019; Hul et al., 2021; Ramser et al., 2021, 2022), since the highest incidences of this condition occur at these ages (McNamee and Smyth, 2000; Paxton et al., 2014; Packialakshmi et al., 2015). Therefore, molecular mechanisms involved with femoral head separation in young age (21 days) broilers were identified using RNA sequencing, allowing highlighting genes and BP potentially related to the onset of this condition.

## Material and methods

### Experimental animals and sample collection

Cobb500 male broilers used in this study were raised in a standard system for commercial growing broilers with the management conditions following the recommendations for this line, with water and feed supplied *ad libitum*. At 21 days of age, 15 lameness and 10 normal standard broilers were weighed and euthanized by cervical dislocation followed by exsanguination, according to the ethical guidelines of the Embrapa Swine and Poultry Ethics Committee on Animal Utilization, under the protocol number 012/2012. Immediately after the slaughter, femurs were evaluated by the presence or absence of FHS, following the methodology described by Paludo et al. (2017). Chickens were considered with FHS when there was separation of the articular cartilage from the growth plate without any visual signal of necrosis, and normal when the AC remained attached to the GP. Samples of one of the femur proximal growth plates were collected individually, immerged in liquid nitrogen and stored at −80°C for further expression analysis.

### Total RNA extraction and quantification

For the RNA-Seq analysis, 4 normal and 4 FHS-affected femur growth plates were chosen and 100 mg of this tissue was homogenized using a mortar with liquid nitrogen. The total RNA was extracted using Trizol (Life Technologies, Carlsbad, United States), according to the manufacturer’s protocol, followed by a RNA cleanup step using the RNeasy mini kit (Qiagen, Hilden, Germany). RNA purity and concentration were measured using Biodrop spectrophotometer (Biodrop, UK, and integrity was evaluated using Bioanalyzer 2,100 (Agilent, Santa Clara, United States). Only samples with RIN >8.0 were considered for further analysis.

### Library preparation and sequencing

Libraries were prepared using TruSeq RNA Sample Prep v2 Kit (Illumina, San Diego, United States ) with 2 μg of total RNA, following the manufacturer´s protocol. The quality of the libraries was evaluated using Bioanalyzer 2,100 (Agilent, Santa Clara, United States ). Libraries were quantified through quantitative PCR using the KAPA Library Quantification kit (KAPA Biosystems, Wilmington, United States ). After pooling the libraries, clustering and sequencing were performed in the Illumina HiSeq 2,500 sequencer (lllumina, San Diego, United States ) producing 2 × 100 bp paired-end reads, at the Functional Genomics Center from ESALQ-USP, Piracicaba, São Paulo, Brazil.

### RNA-seq data analysis

The RNA-seq analysis was performed using BAQCOM, an automated pipeline available at https://github.com/hanielcedraz/BAQCOM (accession on 15th February 2022). Low quality reads (QPhred ≤20), short reads (<70 bp) and Illumina sequence adapters were trimmed using Trimmomatic v0.39 (Bolger et al., 2014). After the quality control, sequence reads of each sample were mapped against the reference chicken genome (GRCg6a, Ensembl release 105) using STAR (Dobin et al., 2013). Reads counting was performed with HTseq-count (Anders et al., 2015), which also generated a count table for statistical analysis. The differentially expressed (DE) genes and heatmap were obtained using the limma package (Ritchie et al., 2015) from the R software (R Core Team, 2021). The Benjamini-Hochberg (BH) methodology (Benjamini and Hochberg, 1995) was applied to correct for multiple testing and genes with false discovery rate (FDR) ≤ 0.05 were considered DE. Negative and positive fold-changes indicate downregulation and upregulation of the genes in the FHS-affected group compared to control group.

### Functional annotation analysis

Functional annotation of DE genes was performed using clusterProfiler (Wu et al., 2021) with MSigDB (Liberzon et al., 2011) and gene ontology with *Gallus gallus* information (org.Gg.eg.db) available at AnnotationDBbi (Pagès et al., 2022) and also in shinyGO v 0.75 (Ge et al., 2020). A search was also performed in the DAVID database, with all genes expressed in the samples used as background, and values of EASE ≤ 0.05 and *p*-values ≤ 0.05 were considered significant. A gene interaction analysis was performed with the STRING database (Szklarczyk et al., 2019) in Cytoscape v 3.9 (Shannon et al., 2003) with *G. gallus* organism using two approaches: 1) only with DE genes and 2) with DE genes plus 5 additional gene interactors.

Additionally, in order to verify the FHS gene expression profile among different ages, Ensembl IDs of DE genes identified in this study were compared with results from transcriptome studies of Peixoto et al. (2019), Oliveira et al. (2020), Hul et al. (2021) and Goldoni et al. (2022). These studies evaluated early stages of FHS at 35 days in growth plate and articular cartilage (Peixoto et al., 2019; Hul et al., 2021; Goldoni et al., 2022) and tibia head separation at 42 days of age (Oliveira et al., 2020).

### qPCR validation

The qPCR analysis was performed with the same 8 GP samples used in the RNA-seq analysis: 4 normal and 4 FHS-affected broilers with 21 days of age. The RNA was extracted as previously described and cDNA synthesis was performed using the SuperScript III First–Strand Synthesis SuperMix kit (Invitrogen, Carslbad, United States ). Primers for *COL14A1, FAM180A, ANGPTL5, FBN2, TNMD*, and *LEPR* candidate genes were used for RNA-Seq confirmation (Table 1). These genes were chosen based on their FDR, logFC, and biological function. Quantitative PCR reactions were performed in the QuantStudio 6 Flex equipment (Applied Biosystems), containing 1 × Mastermix (GoTaq qPCR Master Mix 2×, Promega), 0.16 µM of each F and R primer, 2 µl of cDNA at 1:10 dilution and ultrapure water to complete 15 µl of total reaction. The qPCR reactions were performed in duplicates and the Ct (cycle threshold) values were obtained for differential expression analysis in the REST program (Pfaffl et al., 2002), which uses a nonparametric randomization test to compare the studied groups. For this analysis, Ct means for each sample and gene were used and normalization was performed with *RPL4* and *RPL30* reference genes (Petry et al., 2018). Differential expression was considered significant when *p* ≤ 0.05.

**TABLE 1:** Gene identification and primers used in the qPCR analysis.

Gene	Gene/ensembl ID	Primer squences (5′-3′)
***COL14A1*** ***^a^***	NM_205334.1	F: GTG​ATG​TTG​GAG​CTC​CTG​GT
		R: CAC​ACT​TGA​CGA​GCA​ACA​GC
***FAM180A*** ^**^	ENSGALG00000028459	F:GAGTAGAGCTATGCTTTACCCAGC
		R: CGA​AGC​CAG​CTC​CTC​ATC​TT
***ANGPTL5*** ***^a^***	NM_001197236.1	F: TCA​GGA​AAC​GCA​GGT​GAT​GC
		R: AGT​GCA​CAC​TGG​CCT​ACA​TC
***FBN2*** ^***+***^	ENSGALG00000014686	F: TGC​ATC​GAT​AGC​CTG​AAG​GG
		R: CTA​ATT​CAC​ACC​GCT​CAC​ATG​G
***TNMD***	ENSGALG00000006821	F: CGA​CTA​CAC​GGA​GAA​CGG​G
		R: TGA​TAC​GGC​AGA​TCA​CCC​TG
***LEPR*** ^***++***^	ENSGALG00000011058	F: TGG​TTT​CGC​ACC​GAA​GAA​TG
		R: TTG​CTT​CAG​GGT​GCT​TGA​CA
***RPL4*** ***^a^***	NM_001007479.1	F: TGT​TTG​CCC​CAA​CCA​AGA​CT
		R: CTC​CTC​AAT​GCG​GTG​ACC​TT
***RPL30*** ***^a^***	NM_001007967.1	F: ATG​ATT​CGG​CAA​GGC​AAA​GC
		R: GTC​AGA​GTC​ACC​TGG​GTC​AA

a Petry et al., 2018;^**^ Peixoto et al., 2019, ^+^ Hul et al., 2021; ^++^ Paludo et al., 2017.

### Unmapped read analysis

Part of the reads from RNA sequencing was not mapped in the chicken genome. To verify if those reads could be from microorganisms, the unmapped sequences were submitted to a metagenome taxonomic classification analysis with Metacache software v. 2.2.1 (Müller et al., 2017) with Refseq database version 20200820. Results from family and genera were further processed using the Phyloseq v.1.34.0 package (McMurdie and Holmes, 2013) from R, in which the raw tables were concatenated and only the taxa with more than 100 mapped reads across all samples and present in more than half of the samples were maintained. Abundance analysis was performed using a zero-inflated Gaussian (ZIG) mixed model after cumulative sum scaling (CSS) normalization using the metagenomeSeq package from R (Paulson et al., 2013). Differential abundance of the taxa between groups was considered significant when FDR ≤0.05. Graphics were generated with ggplot2 v. 3.3.3 package from R (Wickham, 2016). Metagenomic classification was registered in the Brazilian National System of Genetic Resources (SISGEN) under ID A62341D.

### Variant identification using RNA-Seq data

RNA sequences obtained in this study, as well as sequences from other GP and cartilage from normal and FHS-affected samples from our previous studies (Peixoto et al., 2019; Oliveira et al., 2020; Hul et al., 2021; Goldoni et al., 2022) were used to verify the presence of variants involved with FHS. To this, sequences from femoral GP and AC of 35-day-old and tibia GP from 42-day-old broilers were downloaded from SRA database bioprojects # PRJNA352962, PRJNA350521, and PRJNA352716, respectively, totaling 24 samples (9 normal and 15 FHS-affected). Sequences were trimmed as described in the RNA-Seq analysis section, and mapping against the chicken reference genome (GRCg6a, Ensembl release 105) was performed using the two pass-mode in STAR software (Dobin et al., 2013). Variant identification was performed using the Genome Analysis Tool kit 3.6 (GATK), following the best practices guidelines for transcriptome variant analysis. Firstly, the genome index, read groups assignment and marking duplicates were performed using Picard tools 2.5 (https://broadinstitute.github.io/picard/index.html). The GATK was used for CIGAR string determination (SplitNCigarReads), reassigning mapping qualities, base recalibration, variants calling and filtering. The following filters were used to select variants: FS > 30.0, MQRankSum < −12.5, SNPcluster considering 3 variants in a 35bp window, QD < 5.0, MQ < 50.0, GQ < 5.0, QUAL ≥ 30.0, ReadPosRankSum < −8.0 and DP ≥ 300.0. Once the polymorphisms were identified by GATK, they were submitted to quality control analysis in plink 1.9 (Purcell et al., 2007; Steiß et al., 2012), where SNPs with genotyping rate <0.2 and minor allele frequencies (MAF) < 0.05 were removed from further analysis. Then, a case-control association analysis using permutation was performed to verify the presence of variants related to FHS phenotype. *p*-values ≤ 0.0001 were considered significant. Variant annotation was performed in Variant Effect Predictor (VEP) tool (McLaren et al., 2016) from Ensembl using the *G. gallus* genome (GRCg6a, Ensembl 105), considering the distance of 1,000 bp for upstream/downstream transcript assignment.

## Results

### Sequencing and mapping

RNA-Seq data from femoral head of all 8 samples generated approximately 22 million paired-end reads (2 × 100 pb) per sample. After the quality control, about 20.3 million of paired-end reads (Supplementary Table S1) were kept for further analysis. An average of 95.81 ± 0.27% (about 19.5 million) of the reads were mapped in the reference chicken genome (GRCg6a, Ensembl 105), where 77.58% of the reads were mapped in features.

### Differential expression, annotation, and pathway analysis

**FIGURE 1:**
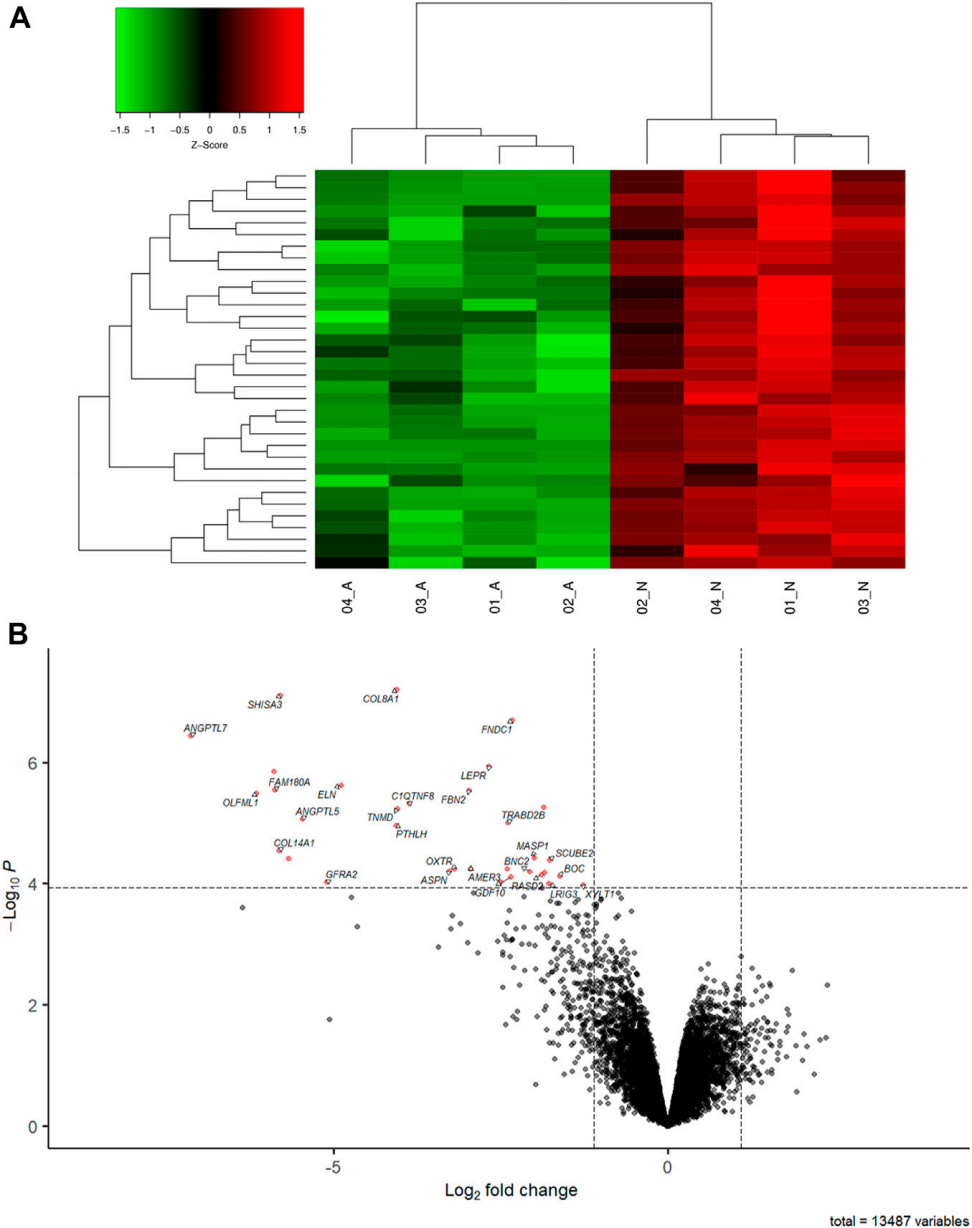
**(A)** Heatmap showing a hierarchical clustering of genes and samples of the DE genes between FHS-affected and control. The genes are presented in the rows and samples in the columns. Downregulated genes in the affected group are shown in red and upregulated in green. **(B)** Volcano plot showing the differentially expressed genes (red dot) in FHS-affected broilers.

The clustering of samples using the DE genes highlight the differences between FHS and normal groups (Figure 1A). 13,487 genes (Supplementary Table S3) were expressed in the 21 days chicken femoral head transcriptome, corresponding to almost 55.40% of the total genes described in the *G. gallus* assembly GRCg6a*.* Out of those, 34 were differentially expressed (DE; Table 2) between the normal and FHS-affected broilers analyzed in this study. All of them were downregulated in the affected (Figure 1B) compared to the normal group.

**TABLE 2:** Differentially expressed genes downregulated in FHS-affected broilers, according to FDR.

T	Gene name	Gene description	LogFC	FDR
ENSGALG00000015253	*COL8A1*	collagen type VIII alpha 1 chain	−4.06	0.0005
ENSGALG00000045153	*SHISA3*	shisa family member 3	−5.79	0.0005
ENSGALG00000011663	*FNDC1*	fibronectin type III domain containing 1	−2.33	0.0009
ENSGALG00000003179	*ANGPTL7*	angiopoietin like 7	−7.14	0.0012
ENSGALG00000011058	*LEPR*	leptin receptor	−2.67	0.0031
ENSGALG00000049529			−5.90	0.0032
ENSGALG00000014686	*FBN2*	fibrillin 2	−2.97	0.0043
ENSGALG00000028459	*FAM180A*	family with sequence similarity 180 member A	−5.88	0.0043
ENSGALG00000032220	*ELN*	elastin	−4.88	0.0043
ENSGALG00000027184	*OLFML1*	olfactomedin like 1	−6.15	0.0044
ENSGALG00000005253	*C1QTNF8*	C1q and tumor necrosis factor related protein 8	−3.87	0.0059
ENSGALG00000006821	*TNMD*	tenomodulin	−4.05	0.0060
ENSGALG00000054999			−1.86	0.0060
ENSGALG00000017191	*ANGPTL5*	angiopoietin like 5	−5.47	0.0082
ENSGALG00000027655	*TRABD2B*	TraB domain containing 2B	−2.40	0.0088
ENSGALG00000017295	*PTHLH*	parathyroid hormone like hormone	−4.06	0.0092
ENSGALG00000037675	*COL14A1*	collagen type XIV alpha 1 chain	−5.82	0.0223
ENSGALG00000007419	*MASP1*	mannan binding lectin serine peptidase 1	−2.00	0.0273
ENSGALG00000015307	*ABI3BP*		−5.67	0.0273
ENSGALG00000032161	*SCUBE2*	signal peptide CUB domain and EGF like domain containing 2	−1.77	0.0273
ENSGALG00000003138	*OXTR*	oxytocin receptor	−3.20	0.0331
ENSGALG00000041501	*AMER3*	APC membrane recruitment protein 3	−2.96	0.0331
ENSGALG00000045154			−2.40	0.0331
ENSGALG00000004722	*ASPN*	biglycan	−3.27	0.0333
ENSGALG00000015101	*BNC2*	basonuclin 2	−2.07	0.0333
ENSGALG00000026836	*COL16A1*	collagen type XVI alpha 1 chain]	−1.84	0.0333
ENSGALG00000012542	*RASD2*	RASD family member 2	−1.90	0.0358
ENSGALG00000015152	*BOC*	BOC cell adhesion associated oncogene regulated	−1.62	0.0360
ENSGALG00000005985	*GDF10*	growth differentiation factor 10	−2.35	0.0361
ENSGALG00000032856	*GFRA2*	GDNF family receptor alpha 2	−5.10	0.0410
ENSGALG00000054344			−2.51	0.0410
ENSGALG00000009755	*LRIG3*	leucine rich repeats and immunoglobulin like domains 3	−1.78	0.0418
ENSGALG00000006757	*XYLT1*	xylosyltransferase 1	-1.27	0.0428
ENSGALG00000012362	*THSD7B*	thrombospondin type 1 domain containing 7B [	-1.88	0.0463

Considering the DE genes, it was possible to observe 33 coding genes, being 29 known, 4 novel and 1 lncRNAs (ENSGALG00000045154). The sequences of 4 uncharacterized genes had similarities with fibronectin type III (ENSGALG00000015307, ENSGALG00000049529), immunoglobulin-like domain (ENSGALG00000054344), and concanavalin A-like lectin domain superfamily (ENSGALG00000054999).

**FIGURE 2:**
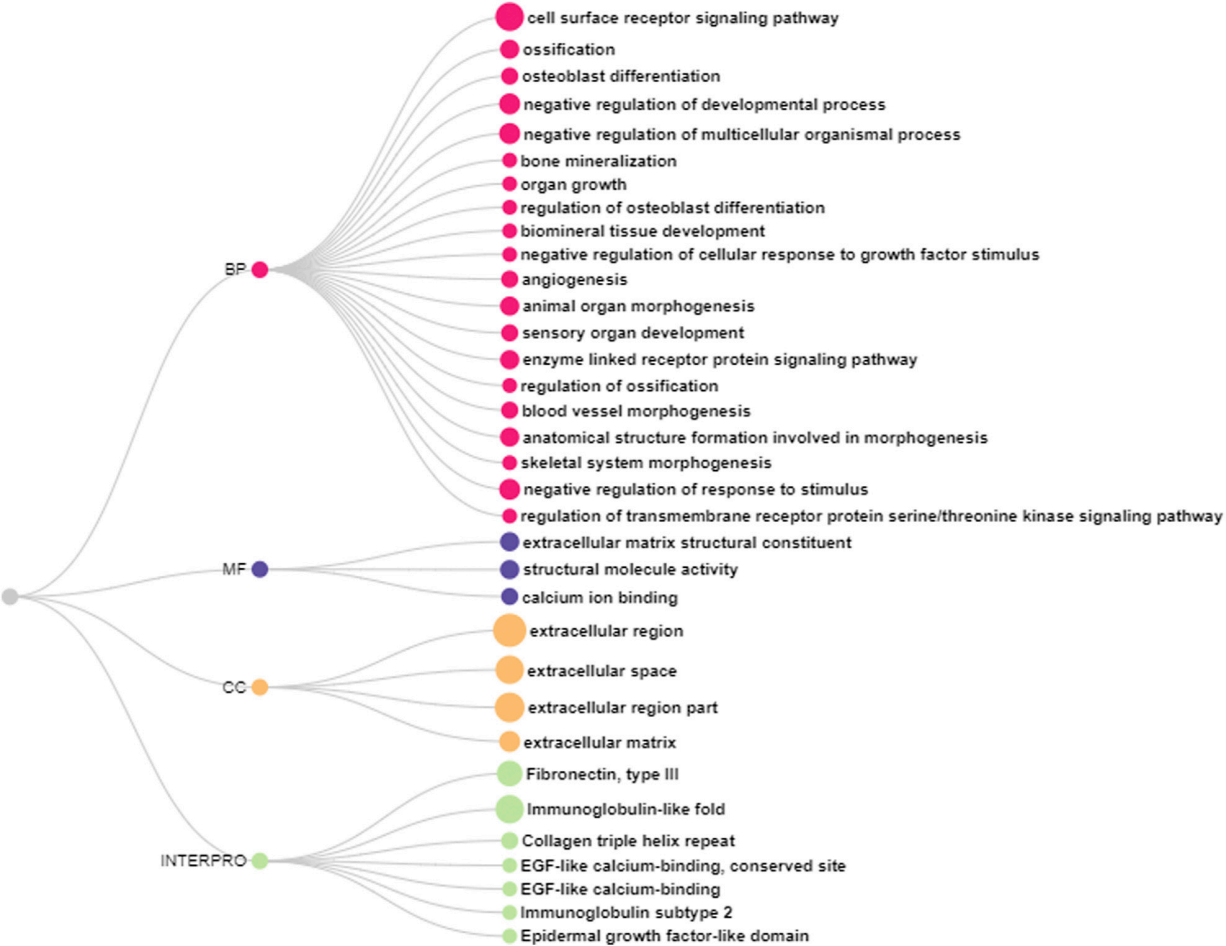
Biological processes (BP), molecular functions (MF), cell component (CC) and protein domains according to the Interpro enriched using DAVID database.

In the DAVID ontology analysis, 20 genes (*ANGPTL7, ASPN, GDF10, TNMD, LRIG3, LEPR, FBN2, BNC2, BOC, COL8A1, PTHLH, TRABD2B, SCUBE2, GFRA2, AMER3*, and *SHISA3*) were attributed to biological processes, where most of them were related to receptor signaling pathways, ossification, bone mineralization and formation, and vascularization (Figure 2; Supplementary Table S4). Using the ShinyGO tool, *ANGPTL7, XYLT1, MASP1, FBN2, PTHLH, SCUBE2*, and *ELN* genes were in the extracellular region BP. The molecular functions were mostly related to calcium ion binding and matrix constituent (Figure 2, Supplementary Table S4). Furthermore, these genes were characterized in fibronectin, collagen, immunoglobulin, and epidermal growth factor protein domains (Figure 2).

**FIGURE 3:**
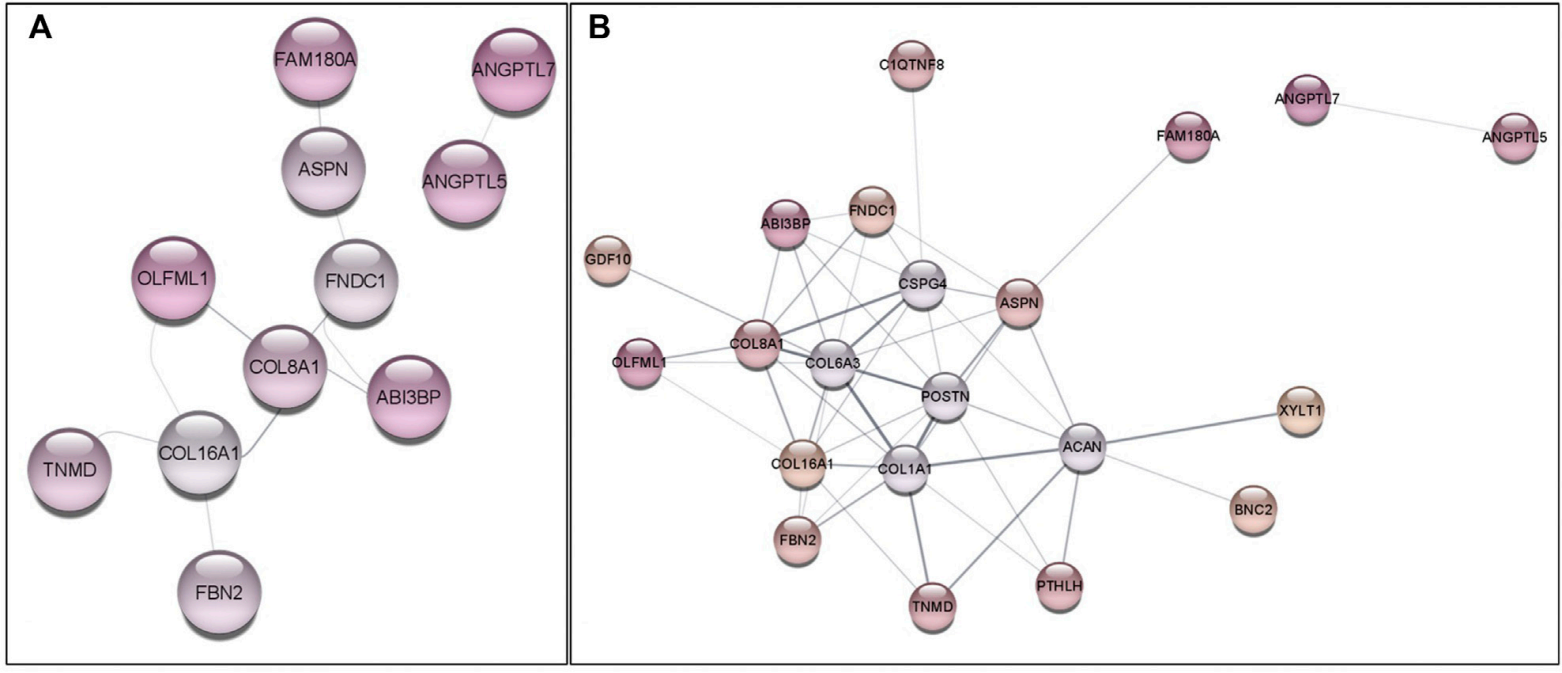
Gene network constructed with DE genes **(A)** and with DE genes plus up to 5 protein interactors **(B)** with the Gallus gallus information. Colored circles represent genes and connecting lines represent interactions among genes, according to Cytoscape Stringdb App. Colors indicate the downregulation expression levels, where darker colors have lower logFC levels in FHS-affected group. Gray circles **(B)** are protein interactors added to improve the gene network information.

The gene network using *G. gallus* database was performed to verify the interactions among the DE genes, where 11 genes were grouped in two main branches (Figure 3A). Considering the gene network with the protein interactors, also 2 main branches were found (Figure 3B). Furthermore, 16 out of 34 DE genes were included in the gene network, being possible to observe that these genes are functionally associated, contributing to FHS phenotype.

**FIGURE 4:**
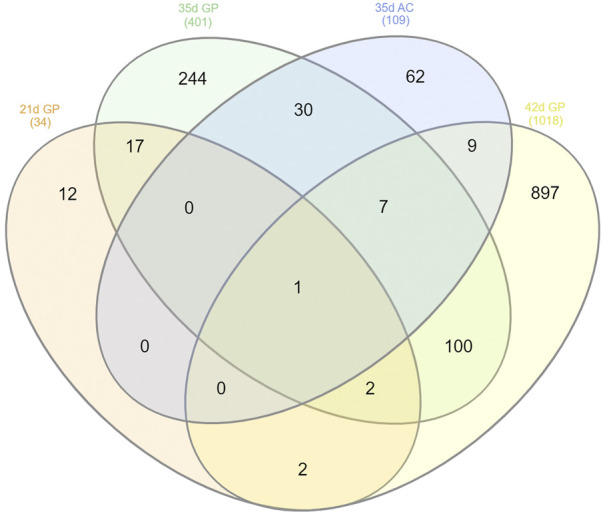
Venn diagram of DE genes in the femur GP of 21 and 35 days of age, tibia GP of 42 days of age and AC of 35 days of age.

Considering the transcriptome datasets from 21, 35, and 42 days of age, it was possible to observe that one gene was DE at all ages in the articular cartilage and growth plate (*FBN2*), while three genes (*FBN2, C1QTNF8* and *XYLT1*) where DE in GP at 21, 35, and 42 days of age (Figure 4). A total of 12 genes were exclusively DE at 21 days, where 10 have already been characterized (*SHISA3, FNDC1, ANGPTL7, LEPR,* ENSGALG00000049529, *OXTR,* ENSGALG00000045154, *COL16A1, RASD2, BOC, GDF10*, and *THSD7B*).

The genes evaluated in the qPCR analysis had the same expression profile when compared to the RNA-Seq results and were also DE between groups. The *COL14A1, FAM180A, ANGPTL5, FBN2*, and *LEPR* candidate genes were downregulated in the FHS-affected when compared to normal broilers, confirming the RNA-Seq analysis results (Table 3). The *TNMD* gene had a low expression, showing amplification (Ct mean = 36.7) in all normal samples, but it was detected in only one sample from the FHS-affected group (Ct mean = 39.2), therefore, it was not possible to perform the statistical analysis for this gene.

**TABLE 3:** Relative gene expression and LogFC between FHS-affected.and control groups and results of differential expression analysis using REST.

Gene	Relative expression	Std. Error	95% C.I.	*p*-value	LogFC
*Col14A1*	0.015	0.007–0.035	0.004–0.049	0.01	−6.06
*FAM180A*	0.005	0.003–0.009	0.002–0.017	0.017	−7.64
*ANGPTL5*	0.015	0.007–0.027	0.004–0.047	0.001	−6.06
*FBN2*	0.396	0.245–0.649	0.192–0.931	0.03	−1.34
*LEPR*	0.131	0.098–0.176	0.076–0.214	0.001	−2.93

### Variant identification

A total of 223,069 variants were identified in the 24 analyzed samples after GATK filtering. For association analysis, 8,316 and 45,615 variants were removed due to genotyping rate and MAF failures, respectively. A total of 168,672 SNPs were kept for association analysis, where 12 SNPs were associated with FHS (*p* < 0.0001). From those, 5 were novel and 7 were existing variants being located in 7 genes (*RARS, TFPI2, TTI1, MAP4K3, LINK5*,*4* and *AREL1)* (Table 4, Supplementary Table S5). The SNP located in the *TTI1* gene was a missense variant, while those in *RARS, TFPI2, LINK54*, and *AREL1* were synonymous. One SNP in the *MAP4K3* gene was located in an intron and three (4:46318416, 4:82213680 and 4:82216997) were in intergenic regions (Supplementary Tables S5, S6).

**TABLE 4:** SNPs associated with FHS in broilers.

SNP location	Gene symbol	Minor allele	Frequency of allele in cases	Frequency of allele in controls	Major allele	*p*-value	Odds ratio	Empirical *p*-value
2:23307098	*TFPI2*	A	0.03333		C	0.0000308	0.02759	0.00004691
3:16824258	*MAP4K3*	C	0.03333	0.4444	G	0.0004111	0.0431	0.00005369
4:46304089	*LIN54*	T	0.5333	0	A	0.0001478	NA	0.0000995
4:46315234	*LIN54*	A	0.5333	0	G	0.0001478	NA	0.0000995
4:46318356	—	A	0.5333	0	G	0.0001478	NA	0.0000995
4:46318416	—	C	0.5333	0	T	0.0001478	NA	0.0000995
4:82213680	—	G	0.1667	0.7222	A	0.0001186	0.07692	0.000092
4:82216997	*GRK4*	C	0.1667	0.7222	T	0.0001186	0.07692	0.000092
5:38228766	*AREL1*	A	0.03333	0.5625	G	0.0000341	0.02682	0.0000281
13:5487116	*RARS*	C	0.1667	0.7778	G	0.00002772	0.05714	0.00005413
13:5487611	*RARS*	G	0.1667	0.7778	A	0.00002772	0.05714	0.00005413
20:10404280	*TTI1*	A	0.6071	0.1111	G	0.0008542	12.36	0.00003559

### Unmapped reads identification

An average of 605,218 RNA reads/sample were unmapped in the chicken genome (Supplementary Table S2) and were submitted to a metagenome taxonomic classification analysis to check if they matched with microorganisms sequences. About 23,608 (∼3.8%) reads/sample were identified in 23 different families (Supplementary Table S7) where the most abundant were *Enterobacteriaceae, Comamonadaceae* and *Bacillaceae*, (Supplementary Table S7). Considering genus, 83 were identified and the most present were *Streptomyces, Pseudomonas, Proteus, Halomonas, Rhizobium, Stenotrophomonas, Shapirovirus, Corynebacterium, Acinetobacter*, and *Aeromonas* (Supplementary Figure S1). Although several groups were found in the analyzed samples, no differences were observed between normal and FHS-affected groups.

## Discussion

The complexity of the FHS/FHN etiology and pathogenesis makes this condition one of the main problems affecting fast-growing broilers production (Prisby et al., 2014). FHS is characterized by the detachment of GP and AC, occurring in tibia and femur (Durairaj et al., 2009; Packialakshmi et al., 2015). Although some studies have been published in the last years with FHS, FHN, and BCO, including by our group, there are few of them approaching the molecular mechanisms involved with these conditions, especially with early ages (Li et al., 2015; Paludo et al., 2017; Peixoto et al., 2019; Oliveira et al., 2020; Liu K. et al., 2021; Hul et al., 2021; Ramser et al., 2021, 2022; Goldoni et al., 2022; Santos et al., 2022). To the best of our knowledge, this paper is the first attempt to compare normal and FHS-affected femoral head transcriptome in broilers with 21 days of age. The results presented here can contribute to deepen the understanding of FHS in chicken, clarifying BP, genes and variants involved with this condition, as well as to prospect the presence of microorganisms in the analyzed samples. Here, most of the enriched biological processes were related to ossification, developmental processes, response to stimulus, extracellular matrix (ECM), angiogenesis, skeletal morphogenesis, within others. In these processes, collagen, angiopoietin-like, growth factors, fibronectins and tumor necrosis related genes were DE (Table 1). The skeletogenesis is initiated when mesenchymal precursor cells differentiate in cartilage and bone cells that grow by the endochondral ossification process (Goldring et al., 2006; Ağirdil, 2020). After hatch, mesenchymal cells in the GP initiate a proliferation to hypertrophic chondrocytes leading to cartilage formation, where chondrocytes will suffer chondrolysis and apoptosis, allowing vascular invasion and bone formation (Rath and Durairaj, 2022). The coordinated gene expression related to cell proliferation and differentiation, angiogenesis, apoptosis, local growth factors, hormones and cell signaling are needed (Goldring et al., 2006). In our study, 34 genes were DE between normal and FHS-affected 21 days old broilers, being downregulated in the affected group (Figure 1B; Table 2). Most of these genes were in BP related to ossification, chondrogenesis and angiogenesis (Figure 2), and the DE profile found in our study could help to understand the FHS pathogenesis. FHS has been characterized by focal death, atrophic changes in the cartilage (Packialakshmi et al., 2015), reduced chondrocyte density, presence of pyknotic nuclei and erythrocytes and the presence of inflammatory cells (Wilson et al., 2019). Here, several genes DE, such as collagens, *FBN2, ANGPTLs, FNDC1, C1QTNF8*, and *XYLT1* may corroborate with the hypothesis suggested by those authors.

GP zones have high metabolic activity, where chondrocytes pass from resting, proliferative, hypertrophic and terminal phases, leading to a healthy osteogenesis (Ağirdil, 2020). In the first phases, it is known that collagens such as *COL2A1* and *COL10A1* are essential to chondrocyte maturation (Ağirdil, 2020). The major constituent of the organic bone matrix and articular cartilage is the collagen protein, where the *COL2A1* is the main studied collagen (Hallett et al., 2019). This protein interacts with other collagens and proteins to create a network to form the ECM (Graham et al., 2000; Flowers et al., 2017), being highly expressed in the proliferation phase, while type X collagens are more expressed in the hypertrophic phases than in the other ones. In our study, three collagen genes, *COL14A1, COL8A1*, and *COL16A1*, were downregulated in the affected broilers, being, respectively, 7, 4, and 6.5 times less expressed in the FHS-affected than in the normal broilers (Table 1).

Furthermore, other collagen related genes, such as *FBN2* and *COL16A1* were also downregulated in the affected group. It is interesting to note that most of the genes in the network were grouped in one branch, where collagen genes were connectors (Figure 3). Although the endochondral ossification has been widely studied (Long and Ornitz, 2013), information on the genes involved in the endochondral ossification and FHS in chickens are still scarce. *COL14A1* has already been associated to calcium ligation and cell morphogenesis (Rojas-Peña et al., 2014), being highly expressed when submitted to mechanical stress (Manon-Jensen and Karsdal, 2016). *COL16A1* was associated to osteoarthritis in humans (Karlsson et al., 2010; Chou et al., 2013), while polymorphisms in *COL8A* gene were associated to the loss of function during embryogenesis leading to congenital vertebral malformations (Gray et al., 2014). The importance of *COL2A1* in the ECM, cartilage and bone replacement formation has been highlighted in several studies. Polymorphisms in this gene were found to increase the susceptibility to FHN in humans (Li et al., 2014), and its expression is essential to the fibrils formation on epiphyseal growth plate (Pouya and Kerachian, 2015). The downregulation of these collagen genes may influence the extracellular matrix formation, affecting the integrity of the collagen network (Heuijerjans et al., 2017), as well as the chondrocyte maturation, and, possibly, preventing the bone formation and, consequently, facilitating the FHS appearance in chickens.

Lack of angiogenesis and vascularization have also been associated to FHS in chickens (Durairaj et al., 2009; Prisby et al., 2014; Li et al., 2015; Packialakshmi et al., 2015; Paludo et al., 2017; Peixoto et al., 2019; Goldoni et al., 2022) and the DE genes *ANGPTL7, TNMD, LEPR*, and *COL8A1* were in BP related to these functions. The angiopoietin-like family is composed by 8 genes (*ANGPTL1* to *ANGPTL8*) encoding proteins structurally similar to angiopoietins, with functions related to glucose metabolism, hematopoiesis, fat metabolism and inflammation, participating in a multitude of physiological and pathophysiological processes. *ANGPTLs* differ from angiopoietins because they do not bind to their receptors (Santulli, 2014). In our study, 2 out of 8 known angiopoetin-like were DE between groups: *ANGPTL7* and *ANGPTL5*. The role of *ANGPTLs* in vascularization still needs to be better understood, since they may act as pro-angiogenic, anti-angiogenic and also VEGF-independent (Hato et al., 2008). *ANGPTL7* and *ANGPTL5* have a role in hematopoietic stem cell expansion (Zhang et al., 2006). *ANGPTL7* is believed to be a potent target of the WNT/β-catenin signaling pathway, which is essential to BMPs (bone morphogenetic proteins) activation in osteoblasts (Beederman et al., 2013; Osório, 2015). Moreover, the alteration of *ANGPTL7* influences the expression of several matrix proteins, such as fibronectin, collagens, myocillins and metalloproteinases (Santulli, 2014), encoded by genes that were also downregulated in our study and were grouped in enriched BP of ECM. The *TNMD* function is not well characterized, but this gene encodes a chondromodulin-I related protein, which increases chondrocyte growth and inhibits angiogenesis (Shukunami et al., 2005; TNMD, 2022). Another downregulated gene in broilers with 21 days of age was *LEPR,* which is involved with lipid metabolism and inflammatory response (Abete et al., 2009; Gogiraju et al., 2019). Mutations in this gene has already been associated to femoral head osteonecrosis in humans (Liu T. et al., 2021) and with bone integrity traits in broilers (Ibelli et al., 2014). Although there is no information on its role associated with FHS in chickens, in mice, *LEPR* was considered a negative modulator of bone mechanosensitivity, leading to a poor osteogenic response (Kapur et al., 2010).

The correct transportation of transmembrane adhesion molecules is important to maintain the articular cartilage and growth plate communication and, consequently, bone remodeling (Packialakshmi et al., 2015). In the current study, low expression levels of genes associated with ECM could favor the FHS condition. Among the genes DE in BP related to extracellular matrix were *ASPN, FBN2, COL8A1, COL14A1, COL16A1,* and *ELN.* The *ELN* was downregulated in FHS-affected broilers compared to normal ones in this study and in broilers with 35 days of age (Peixoto et al., 2019). This gene is associated with the production of a protein called tropoelastine, which is responsible for the elasticity of connective tissue found in cartilage, and acts as a precursor of osteoblast differentiation (Twine et al., 2014). Reduced *ELN* expression was associated to the risk of injury in the rat tendon (Kostrominova and Brooks, 2013).The A*SPN* gene encodes a small leucine-rich cartilage extracellular protein of proteoglycan family, regulating chondrogenesis and binding calcium to collagens (Mishra et al., 2019). A*SPN* is considered a biological marker for osteoarthritis development in humans and mice (Karlsson et al., 2010; Mishra et al., 2019). This gene also acts in osteoblast biomineralization activity, and its expression was increased in ECM (Zhu et al., 2012).

FHS occurrence is also affected by broilers age, increasing at older ages (Prisby et al., 2014). Our research group has been evaluating the FHS/FHN gene expression in several ages in femur (Paludo et al., 2017; Hul et al., 2021; Goldoni et al., 2022; Santos et al., 2022) and in tibia (Oliveira et al., 2020). In this way, trying to understand the genes more related with FHS onset, we have compared the common DE genes and also those exclusive to 21, 35, and 42 days of age in GP and AC. The *FBN2* was the only DE gene in all studied datasets: 21 and 35 days femur GP, 42 days tibia GP and 35 days AC (Figure 4; Supplementary Table S8). This gene was annotated in 23 of the 34 ontologies enriched in this study (Figure 2; Supplementary Table S4), including ossification, regulation of TGF, extracellular matrix and calcium binding. *FBN2* gene is one of the major components of ECM with a primary function of maintaining tissue structural integrity, which can affect several tissues, including skeletal system (Yu and Urban, 2013; Lee et al., 2019). In *FBN2* knockout mice, a reduced osteoblast maturation was observed, preventing bone formation, and increasing bone resorption (Nistala et al., 2010), showing that this gene has an important role in stimulate osteoblast differentiation (Lee et al., 2019). In dogs, mutations in *FBN2* gene were associated with hip dysplasia (Friedenberg et al., 2011). In the analyzed datasets, the *FBN2* downregulation in the FHS-affected group could be preventing the optimal ossification of the GP and, since it occurred at 21, 35, and 42 days of age, this reinforces *FBN2* gene as candidate for FHS/FHN.

We also observed that three genes (*FBN2, XYLT1*, and *C1QTNF8*) were downregulated in GP of FHS-group of all analyzed ages. *XYLT1* encodes the xylosyltransferase enzyme, necessary for glycosaminoglycan (GAG) biosynthesis, and it is considered a key gene for chondrocyte maturation and skeletal length (Mis et al., 2014). In mice, *XYLT* anomalous expression, as well as mutations in this gene alters the GAG normal levels, affecting proteoglycan production and bone growth, leading to a pug dwarfism phenotype (Mis et al., 2014). Furthermore, it was demonstrated in zebrafish that bone formation around cartilage is space and time regulated, in which proteoglycans were responsible for this regulation, being crucial for skeletal development (Eames et al., 2011). There is a lack of information on the *C1QTNF8 (*complement C1q Tumor Necrosis Factor-Related Protein 8) gene function in chickens. *C1QTNF8* is predicted to be part of a collagen trimmering and, in humans, it is considered a paralog of *C1QTNF6*. A DNA methylation analysis found that the *C1QTNF8* was differentially methylated in osteoarthritis (OA) subchondral bone (Jeffries et al., 2016), while its paralogous is a marker for OA in mouse, acting in host defense, inflammation and glucose metabolism (Murayama et al., 2014, 2015). Moreover, when comparing DE genes in GP from different ages, we observed that through aging, more shared DE genes were found between 21 and 35 days and between 35 and 42 days of age, than when 21 and 42 days were compared (Figure 4). When looking at the 12 exclusively DE genes between normal and FHS-affected broilers at 21 days of age (*SHISA3, FNDC1, ANGPTL7, LEPR, OXTR, COL16A1, BOC, E*NSGALG00000049529, *RASD2, GDF10* ENSGALG00000045154, and *THSD7B)*, they were mainly related to WNT and FGF signaling (Amanatullah et al., 2014), bone metabolism (Nilsson et al., 2007; Di Benedetto et al., 2014; Lui et al., 2018), angiogenesis (Shang et al., 2015; Huang et al., 2021), chondrogenesis (Sekiya et al., 2002; Paradise et al., 2018) and Hedgehog signaling pathway (Kavran et al., 2010; Xavier et al., 2016). In this way, it is possible to highlight that DE genes between normal and affected broilers at 21 days of age were mainly related to endochondral ossification, while when GP and AC at 35 and 42 days of age were evaluated (Peixoto et al., 2019; Oliveira et al., 2020; Hul et al., 2021; Goldoni et al., 2022), there were several DE genes related to other biological processes, such as inflammation, defense response, chemotaxis, within others. Therefore, the expression profiles found at 21 days of age are in agreement with other studies that found that one of the main issues regarding FHS/FHN is related to the femur maturation and mineralization (Prisby et al., 2014; Wilson et al., 2019), possibly due to fast-growth and lack of increasing bone volume in the same rate.

Variants in all datasets analyzed here were identified in the RNA-Seq and an odds ratio analysis was performed. Twelve SNPs in seven genes (*TFPI2, MAP4K3, LIN54, GRK4, AREL1, RARS*, and *TTI1*) were associated with FHS predisposition in chickens (*p* < 0.0001). A new SNP annotated as missense in the TELO2 Interacting Protein (*TTI1)* gene had an odds ratio of 12.36. The *TTI1* is an mTOR signaling that regulates cell growth and survival in response to nutrient and hormonal changes and activates the eukaryotic translation initiation factor 4E binding protein 1 (*eIF4E*) (Ronkina and Gaestel, 2022) from 4E-BP1, which is an inhibitor of cap-dependent translation (Katsara and Kolupaeva, 2018). The 4E-BP1 inhibition has already been associated to cartilage degeneration in rat osteoarthritic knees (Katsara and Kolupaeva, 2018). The other variants were intronic or synonymous, but it is interesting to note that, as the missense, they were found in genes mainly related to basal machinery of cells, such as *RARS1*, *LIN54, AREL1*, and *TFPI2. RARS1* is an Arginyl-TRNA Synthetase 1, *LIN54* is a key regulator of cell cycle genes, in which its depletion leads to growth defects (Schmit et al., 2009), the *AREL1* is a negative regulator of apoptosis, while *TFPI2* is related to vascular endothelial growth factor (Xu et al., 2006). Although with a small number of samples, the RNA-Seq data allowed us to identify putative functional variants that could contribute with the appearance of the analyzed condition. The SNPs discovered here should be validated in a large population to confirm them as genetic markers.

According to our results, several biological processes and genes involved with femur head separation in chickens were identified at an early age. Here, genes of the collagen and angiopoietin-like family, among others, were associated with this condition in *G. gallus* at 21 days of age. It was possible to show that several genes, such as *FBN2, XYLT1*, and *C1QTNF8* have an important role in the organic matrix bone formation, hypoxia, as well as its homeostasis. This indicates that changes associated with broiler rapid growth may affect genes related to osteogenesis, especially those involved with endochondral ossification, which might contribute to the onset of femoral head necrosis. Although it is difficult to state whether these genes are causing FHS or not, the approach used in this study allows comprehending how the early molecular changes are happening. Nevertheless, further studies are needed to clarify the expression pattern of these genes over time, to elucidate whether the alteration in expression occurs since the birth of the chicks, or if it is due to their rapid growth. Moreover, SNPs in candidate genes were prospected in the femoral GP of 21-day-old broilers, which can evince new approaches to reduce this condition in chickens. Understanding the genetic factors associated with bone formation and maintenance may lead to a better comprehension on how environmental and management factors can affect the necrosis process.

## Conclusion

In this study, using RNA-seq analysis, we have shown that a set of genes related to chondrogenesis and bone differentiation were downregulated in the GP of FHS-affected young broilers. Among these genes, *FBN2, XYLT1*, and *C1QTNF8* can be highlighted since they were DE in the GP of various ages. SNPs were also identified in genes related to translation factors and cell growth, which could predispose the animals to FHS/FHN development. Furthermore, at 21 days of age, we also notice that the DE genes were more related to cartilage and bone morphogenesis, while in other ages (35 and 42 days), genes related to defense response, inflammation and chemotaxis were also found. Therefore, these findings evince that candidate genes pointed out in our study are probably related to the FHS progression in broilers.

## Data Availability

The datasets presented in this study can be found in online repositories. The names of the repository/repositories and accession number(s) can be found below: https://www.ncbi.nlm.nih.gov/, SRA Bioproject PRJNA288640.
