# Supplementary figures and images for: Downregulation of growth plate genes involved with the onset of femoral head separation in young broilers
Source: Front Physiol. 2022 Aug 8;13:941134. doi: 10.3389/fphys.2022.941134 (PMC9393217; doi:10.3389/fphys.2022.941134)

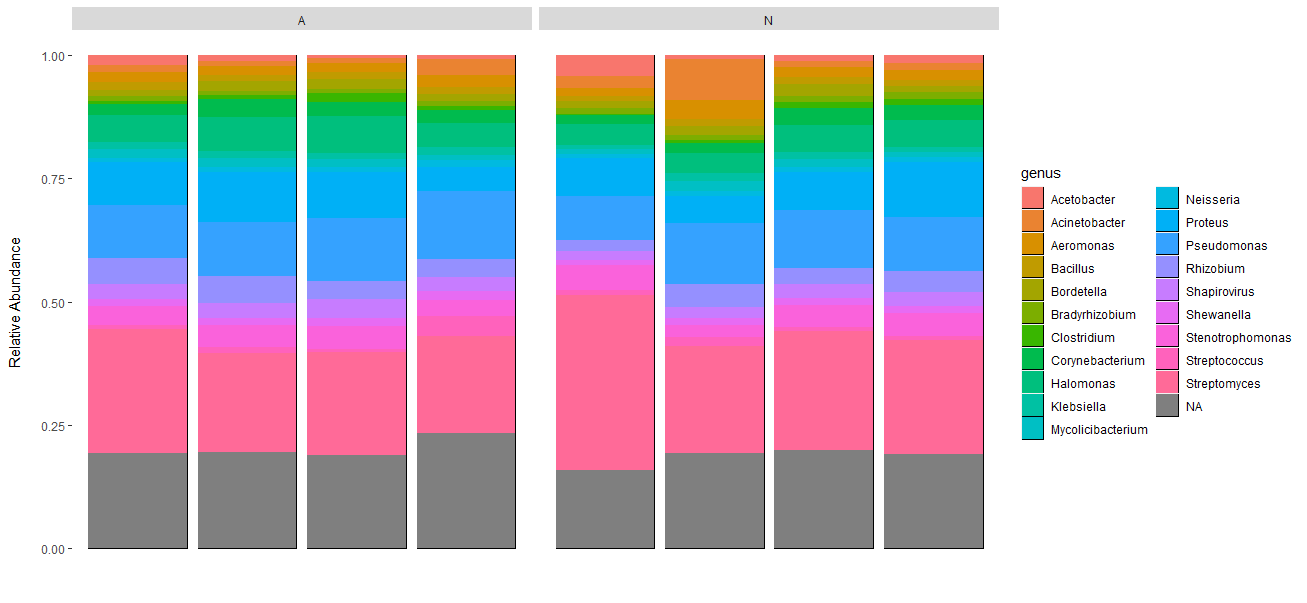

Supplement: Supplementary file 1 [file Image1.TIFF]
